# Epigenetic marks: regulators of livestock phenotypes and conceivable sources of missing variation in livestock improvement programs

**DOI:** 10.3389/fgene.2015.00302

**Published:** 2015-09-28

**Authors:** Eveline M. Ibeagha-Awemu, Xin Zhao

**Affiliations:** ^1^Dairy and Swine Research and Development Centre, Agriculture and Agri-Food CanadaSherbrooke, QC, Canada; ^2^Department of Animal Science, McGill University, Ste-Anne-De-BellevueQC, Canada

**Keywords:** epigenetics, livestock, cattle, sheep, goat, pig, genetic improvement

## Abstract

Improvement in animal productivity has been achieved over the years through careful breeding and selection programs. Today, variations in the genome are gaining increasing importance in livestock improvement strategies. Genomic information alone, however, explains only a part of the phenotypic variance in traits. It is likely that a portion of the unaccounted variance is embedded in the epigenome. The epigenome encompasses epigenetic marks such as DNA methylation, histone tail modifications, chromatin remodeling, and other molecules that can transmit epigenetic information such as non-coding RNA species. Epigenetic factors respond to external or internal environmental cues such as nutrition, pathogens, and climate, and have the ability to change gene expression leading to emergence of specific phenotypes. Accumulating evidence shows that epigenetic marks influence gene expression and phenotypic outcome in livestock species. This review examines available evidence of the influence of epigenetic marks on livestock (cattle, sheep, goat, and pig) traits and discusses the potential for consideration of epigenetic markers in livestock improvement programs. However, epigenetic research activities on farm animal species are currently limited partly due to lack of recognition, funding and a global network of researchers. Therefore, considerable less attention has been given to epigenetic research in livestock species in comparison to extensive work in humans and model organisms. Elucidating therefore the epigenetic determinants of animal diseases and complex traits may represent one of the principal challenges to use epigenetic markers for further improvement of animal productivity.

## Introduction

The goal of animal production is to achieve increased productivity for human purposes while enhancing health and wellbeing of animals. Present gains in livestock productivity have been obtained through intensive genetic selection/breeding and management efforts in the last five decades. Recently, genetic markers associated with improved animal productivity are gaining importance in livestock selection programs. Already, numerous genetic markers, mainly in the form of singly nucleotide polymorphisms and copy-number variations, have been identified and associated with milk production, meat quality, reproduction, and growth traits. Some of these markers are already finding use in genomic selection for these phenotypes (König et al., 2009; Bouquet and Juga, 2013). However, the genomic variation accounted for by DNA markers could only explain a portion of the phenotypic variance in traits (Langevin and Kelsey, 2013). An elusive portion of the genetic variation in animal production traits is likely embedded in the epigenome and remains to be exploited.

Epigenetics and its associated terminologies have several versions of connotations and specific terms need to be defined for further discussions. Between information coded in the genotype and desired phenotypes lies a whole complex of developmental processes for which the term “epigenotype” has been proposed (Waddington, 2012). The term epigenetics since its inception in 1942 has evolved and generally represent heritable states of gene expression that are not dependent on alterations in the DNA sequence. The epigenome of a cell is the complete collection of epigenetic marks, such as DNA methylation, histone tail modifications (acetylation, methylation, ubiquitylation, etc.), chromatin remodeling and other molecules that can transmit information through gene regulation such as non-coding RNA species (e.g., microRNAs and long non-coding RNAs), that exist in a cell at any given point in time (Rakyan et al., 2011). Although the genome of a cell is fairly stable, the epigenome is highly dynamic throughout life and is governed by a complex interplay of genetic and environmental factors (Bernstein et al., 2007). Normal cellular functions rely on the preservation of genetic and epigenomic homeostasis and a dynamic balance of stability and reversibility in gene expression patterns is required to ensure cell identity, maintain growth and development, and enable cells to response to stimuli. A deviation from this balance is highlighted by numerous reported associations between epigenomic perturbations and human diseases, a common example being cancer (Kulis and Esteller, 2010). Epigenetic modifications can be altered by external or internal environmental factors and have the ability to change gene expression and define specific phenotypes. These features advocate epigenetic mechanisms as the missing but yet uncovered regulators in the expression of complex animal traits and disease etiology.

In this review, the current state of knowledge on how nutrition, pathogens and other environmental factors modify epigenetic marks leading to varying effects on reproduction, growth, and production traits in livestock species (cattle, sheep, goat, and pig) will be presented. The potential of applying epigenetic markers to improve productivity will be discussed, as well as the challenges and prospects for advancement.

## Epigenetic Mechanisms

### What They are and What They Do

Epigenetic mechanisms encompass processes that alter gene expression with resultant effects on the phenotype without changes on the DNA sequence, and include DNA methylation, histone tail modifications, chromatin remodeling, and the activities of non-coding RNAs. Epigenetic mechanisms regulate gene expression at the transcriptional and post-transcriptional levels and therefore contribute to phenotypic manifestations. A plethora of information support the regulatory role of epigenetic factors in livestock phenotypes like diseases (Karrow et al., 2011; Luo et al., 2011), reproduction (Urrego et al., 2014) and milk production (Singh et al., 2010).

### DNA Methylation

**FIGURE 1:**
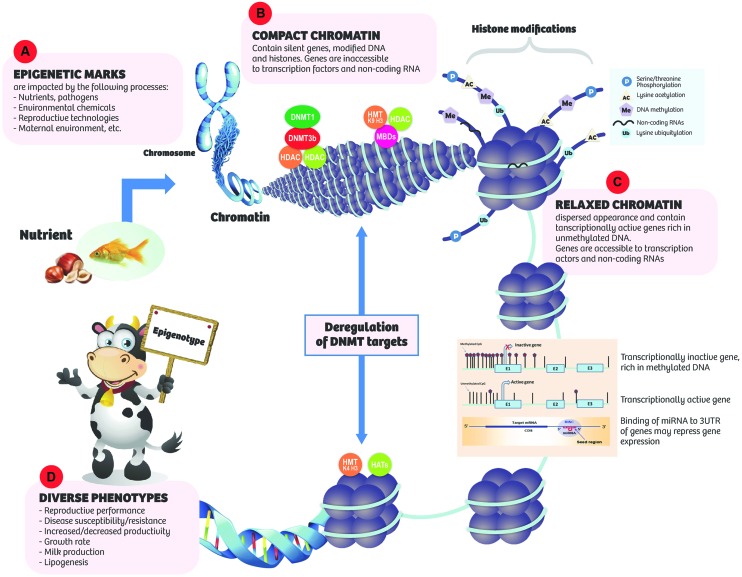
**Epigenetic marks respond to internal and environmental cues **(A)** resulting in various effects on chromatic conformation and gene expression. (B)** Compact chromatin: tends to contain silent genes, modified DNA and histones. A number of nuclear factors such as DNA methyltransferases (DNMTs), methyl-CpG binding domain proteins (MBDs), histone methyltransferases (HMT, K9, H3), histonedeactylases (HDACs), and DNA methylation are involved in silencing gene expression. In the compact state, genes are inaccessible to transcription factors and non-coding RNAs (ncRNAs). **(C)** Relaxed chromatin: has dispersed appearance and is gene rich. Transcriptionally active genes are rich in unmethylated DNA. Histones are generally hyperacetylated. Histone methyltransferases (HMT, K4, H3) and acetyltransferaces (HATs) are associated with unmethylated promoters and transcriptional activity. Genes are accessible to transcription factors and ncRNAs. **(D)** Diverse phenotypes may result.

DNA methylation is a form of epigenetic modification that involves covalent addition of a methyl group to the 5’ position of cytosine base in DNA sequence in a reaction catalyzed by a class of enzymes known as DNA methyltransferases (DNMT1, DNMT3a, and DNMT3b) with *S*-adenosyl-methionine as the methyl donor (Miranda and Jones, 2007). The enzymatic activity of DNMT1 maintains DNA methylation during DNA replication while DNMT3a and DNMT3b are responsible for *de novo* methylation of unmodified DNA. DNA methylation is crucial for genomic stability and is used by mammalian cells to maintain development. DNA methylation occurs mostly at cytosine-phosphate-guanosine (CpG) dinucleotides and to a lesser extent at CpA, CpT, or CpC dinucleotides (Ziller et al., 2011). DNA methylation in the promoter region of genes has been generally associated with transcriptional repression, while their hypomethylation is linked with transcriptional activation leading to increased expression of genes (**Figure 1**). On the other hand, methylation in the body of genes can actually lead to increased transcriptional activation (Langevin and Kelsey, 2013). Therefore, even though all the cells of an organism contain the same genetic information, different tissue/cell types have a unique DNA methylation profile that arises during development and is consequently maintained after DNA replication and cellular differentiation for tissue/cell-specific gene expression.

DNA methylation is the most widely studied epigenetic mechanism of gene regulation. Accumulating lines of evidence indicate that DNA methylation is susceptible to nutritional and environmental influences and alterations in DNA methylation profiles can alter gene expression profiles leading to diverse phenotypes with the potential for increased/decreased productivity and disease risk (Choi and Friso, 2010; Jang and Serra, 2014).

### Histone Modifications

Eukaryotic DNA is tightly packed to form nucleosome, the basic unit of chromatin. The nucleosome is an octamer of four histones (H2A, H2B, H3, and H4) densely packed to achieve a fold compaction necessary to fit a genome into the nucleus while also allowing enough room for proteins involved in transcription, replication, and repair to access DNA (Zentner and Henikoff, 2013). Chromatin may exist in one of two states as follows: (1) euchromatin or relaxed chromatin which is associated with active gene transcription and expression; and (2) heterochromatin or highly compacted and silenced chromatin which is associated with repressed gene expression through hindered access of transcription factors to genes (**Figure 1**). Heterochromatin can be in either constitutive or facultative state. Constitutive heterochromatin comprises mainly repetitive genetic elements, such as telomeres and centromeres and functions as a genome stabilizer that prevents gene rearrangements between highly similar genetic sequences, while facultative heterochromatin is often localized in promoter regions and is established either in a developmentally regulated manner or in response to environmental triggers (Oberdoerffer and Sinclair, 2007).

Generally, the structure of chromatin is under strict regulation by several mechanisms that encompass chromatin remodeling, histone modification, histone variant incorporation, and histone eviction, which imposes major impediments on aspects of transcription mediated by RNA polymerase II (Li et al., 2007). The N-terminal tails of histones are subject to post-translational modifications and today, over 100 distinct histone modifications including lysine methylation, lysine acetylation, serine/threonine phosphorylation, ubiquitination, sumoylation, and crotonylation have been described (Kouzarides, 2007; Tan et al., 2011). Structural changes to chromatin as a result of histone modification usually lead to the recruitment of effector proteins like transcription factors which in turn modulate gene expression. In fact, histone modifications in conjunction with DNA methylation state and specific microRNAs regulate the expression of associated genes (Bernstein et al., 2007). Histone acetylation is associated with active and open chromatin state while lysine methylation at the N-terminal of histones can either repress or activate gene expression depending on the affected lysine residue (Jenuwein and Allis, 2001; Li et al., 2007).

### Non-Coding RNAs

Multiple types of RNAs have been implicated in epigenetic inheritance across generations and include maternal stores of messenger RNAs (mRNAs) and long non-coding RNAs (lncRNAs), as well as various small RNAs [small interfering RNAs [siRNAs], piwi RNAs (piRNAs), microRNAs (miRNA)] that interfere with transcription, mRNA stability, or translation (Mercer and Mattick, 2013; Heard and Martienssen, 2014).

microRNAs are a class of endogenous non-coding small RNAs of about 22 nucleotides in length with capacity to regulate post gene transcription and consequently control the activity of about 60% of all protein coding genes and participate in the regulation of almost every cellular process investigated in mammals (Bartel, 2009; Friedman et al., 2009). miRNAs in animals are processed by the activities of two RNase III-like enzymes, Drosha, and Dicer. A microRNA molecule is synthesized as a long RNA primary transcript known as primary miRNA (pri-miRNA), which is cleaved by Drosha to produce a characteristic stem-loop structure of about 70 base pairs long, known as precursor miRNA (pre-miRNA). A pre-miRNA is cleaved by Dicer to form a mature miRNA. The biogenesis of miRNA has been elaborated upon by several excellent reviews (Filipowicz et al., 2008; Krol et al., 2010). miRNAs by base pairing with mRNA regulate gene expression in animals through four distinct ways: inhibition of translation initiation, inhibition of translation elongation, co-translational protein degradation and premature termination of translation (Huntzinger and Izaurralde, 2011). The ability of miRNAs to regulate gene expression has positioned them as valuable biomarkers or tool for diagnosis of a number of disease conditions in humans including cardiovascular diseases, cancer, neurodegenerative disorders, and infectious diseases (Li et al., 2009; Tüfekci et al., 2014). The role of miRNAs in livestock productivity is beginning to emerge. miRNAs are involve in many processes in farm animals (Wang et al., 2013a) including roles in disease (Karrow et al., 2011; Luo et al., 2011), adipogenesis (Romao et al., 2014), and milk production (Singh et al., 2010).

The number of miRNAs encoded by the genomes of farm animal species (cattle, sheep, goat, and pig) varies considerably from a handful to about 793 in cattle. miRNA detection in livestock species was initially slow but with advances in deep sequencing technologies and computational predictions, the number of miRNAs detected in different livestock species continues to increase. The numbers of miRNA entries in miRBase for cattle, sheep, goat and pig since 2005 are shown in **Table 1**.

**Table 1:** ^∗^Number of microRNAs (miRNAs) reported in miRBase for cattle, sheep, goat, and pig since 2005.

miRBase	Date released	Cattle		Pig		Sheep		Goat	
		^∗∗^miRNA Precursor	Mature miRNA	miRNA precursor	Mature miRNA	miRNA precursor	Mature miRNA	miRNA precursor	Mature miRNA
mirBase 21	June, 2014	808	793	382	411	106	153	267	436
mirBase 20	June, 2013	798	707	208	249	105	104	-	-
mirBase 19	August, 2012	766	682	271	240	55	55	-	-
mirBase 18	November, 2011	662	612	228	210	55	55	-	-
mirBase 17	April, 2011	662	612	228	210	55	55	-	-
mirBase 16	August, 2010	662	612	221	197	4	4	-	-
mirBase 15	April, 2010	665	615	175	163	4	4	-	-
mirBase 14	September, 2009	615	576	77	73	4	4	-	-
mirBase 13	March, 2009	356	322	77	73	4	4	-	-
mirBase 12	September, 2008	117	112	66	64	4	4	-	-
mirBase 11	April, 2008	117	112	55	54	4	4	-	-
mirBase 10.1	December, 2007	117	112	54	53	4	4	-	-
mirBase 10.0	August, 2007	117	112	54	53	4	4	-	-
miRBase 9.2	May 2007	117	112	54	53	4	4	-	-
miRBase 9.1	February 2007	98	97	54	53	4	4	-	-
miRBase 9.0	October 2006	98	97	54	53	4	4	-	-
miRBase 8.2	July 2006	98	97	54	53	4	4		
miRBase 8.1	May 2006	33	33	54	54	4	4	-	-
miRBase 8.0	February 2006	-	-	54	54	4	4	-	-
miRBase 7.1	October 2005	-	-	54	54	4	4	-	-
miRBase 7.0	June 2005	-	-	54	54	4	4		
miRBase 6.9	April 2005	-	-	-	-	-	-	-	-

*^∗^ftp://mirbase.org/pub/mirbase/ retrieved on 20th January 2015. Majority of miRNAs were discovered by next generation miRNA sequencing technologies.*

*^∗∗^Usually, a miRNA precursor is cleaved to give rise to one mature miRNA. In some cases, one or more mature miRNAs could be retained from the same miRNA precursor. Similarly, two or more miRNA precursors from different genomic locations could give rise to the same mature miRNA.*

Long non-coding RNAs are a diverse group of non-coding RNA transcripts >200 nucleotides long with emerging regulatory roles in many biological processes (Mercer et al., 2009; Mercer and Mattick, 2013; Vance and Ponting, 2014). LncRNA make up the largest portion of the mammalian non-coding transcriptome (Mercer et al., 2009). LncRNAs have common biogenesis pathways with mRNAs and other classes of ncRNAs. Majority of lncRNAs are generated by the activities of RNA polymerase II and have a 5′ terminal methylguanosine cap, are often spliced and polyadenylated (Mercer and Mattick, 2013). Alternative pathways have also been shown to contribute to the emergence of a poorly characterized group of non-polyadenylated lncRNAs that are probably expressed from RNA polymerase III promoters (Dieci et al., 2007; Kapranov et al., 2007) or that arise during splicing and small nucleolar RNA production (Yin et al., 2012). Thousands of genes encoding lncRNAs have been identified in mammalian genomes studied so far (Ulitsky and Bartel, 2013; Kapusta and Feschotte, 2014) and about 111,685 human lncRNAs have been annotated (Volders et al., 2014; http://www.lncipedia.org/). In humans, regulatory roles of lncRNA have been associated with several disease conditions including tumorigenesis, cardiac development, aging and immune system development (Esteller, 2011; Atianand and Fitzgerald, 2014; White et al., 2014; Devaux et al., 2015).

Few studies have examined the occurrence and potential functions of lncRNAs in livestock species (Weikard et al., 2013; Billerey et al., 2014; Ibeagha-Awemu et al., 2015). Using RNA sequencing, Weikard et al. (2013) identified a large number (4,848) of potential lncRNAs, predominantly intergenic (4,365), in bovine skin which suggests potential relevance in the regulation of pigmentation processes. In another study, 584 lncRNAs were characterized in bovine muscle in addition to significant correlated expression between 2,083 pairs of lncRNA/protein encoding genes as well as location of some lncRNA genes within quantitative trait loci for meat traits (Billerey et al., 2014). Ibeagha-Awemu et al. (2015) characterized the lncRNA repertoire of the bovine mammary gland by RNA-sequencing and identified 4227 lncRNAs, including 338 known and 3889 novel. Furthermore, they showed that 26 lncRNAs were significantly differentially regulated in response to a diet rich in α-linolenic acid thus suggesting potential regulatory roles of lncRNAs in fatty acid synthesis and lipid metabolism (Ibeagha-Awemu et al., 2015).

### Epigenetic Marks Interact in Function

Accumulating lines of evidence indicate that two or more epigenetic mechanisms may interact to control gene expression. Notably, miRNAs are involved in global DNA hypomethylation through their targeting of DNMTs in 3’untranslated regions of genes. Members of the miRNA-29 family have been shown to revert aberrant methylation in lung cancer by targeting DNA methyltransferases 3A and 3B (Fabbri et al., 2007). miRNA-342 was observed to inhibit colorectal cancer cell proliferation and invasion by directly targeting DNA methyltransferase 1 (Wang et al., 2011). It has also been shown that human miRNAs can induce chromatin remodeling (Kim et al., 2008; Szenthe et al., 2013). In cattle, Wang et al. (2014) showed evidence that miRNA-152 regulates DNA methyltransferase 1 and is involved in the development and lactation processes in mammary glands. Many lncRNAs function as epigenetic modulators by binding to chromatin-modifying proteins thereby recruiting their catalytic activities to specific sites in the genome, and impacting gene expression (Mercer and Mattick, 2013).

## Nutritional Influence on Epigenetics Marks and Effect on Animal Production

A body of evidence suggests that maternal nutritional imbalance, either through global nutritional disproportions or deficiencies in certain nutrients, and environmental exposure during critical developmental periods predisposes to disease susceptibility later in life and a role of epigenetic perturbations has been advocated (DelCurto et al., 2013; Jang and Serra, 2014). A typical example is from the report of the Dutch Famine Birth Cohort Study which showed increased risk of cardiovascular diseases 40–50 years later in children born to mothers who experienced severe malnutrition at the first trimester stage during Nazi occupation from November 1944 to May 1945 (Painter et al., 2006; Roseboom et al., 2006).

Some nutrients, bioactive food components and dietary interventions including high/low fat diets, protein/caloric restrictions, bioactive micronutrients, and plant derivatives have the ability to modify epigenetic marks and alter cellular signaling in the offspring and during growth and development (García-Segura et al., 2013; Guéant et al., 2014; Palmer et al., 2014). Vitamin B-12, folate, choline, betaine, and methionine are nutrients involved in one-carbon metabolism and can alter the methylation state of DNA and histone. In particular, folate (a water soluble B-vitamin) has been extensively studied for its effect on DNA methylation. In cattle, dietary supplementation with a rumen protected B-vitamin complex including folate led to increased conception rate at first service suggesting a link between methylation and conception rate in cows (Juchem et al., 2012).

Dietary fatty acids may also promote establishment of epigenetic marks by stimulating expression of specific genes during critical periods of growth. The peroxisome proliferator activated receptor-α (PPAR-α) transcription factor responds to a diversity of fatty acids to modulate expression of specific genes and PPAR-α facilitated transcriptional activation during critical ontogenic periods may obstruct epigenetic silencing of genes involved in fatty acid metabolism (Waterland and Rached, 2006). Emerging data advocate that lipids and lipoprotein components interrelate directly with chromatin structure to influence gene expression (Davie, 2003; Zaina et al., 2005). Feeding a high-concentrate corn straw diet to dairy cows led to alteration of the methylation state of specific genes involved in fat and protein synthesis in the mammary tissues of dairy cows (Dong et al., 2014). Similarly, supplementing the diets of dairy cows with materials rich in unsaturated fatty acids showed significant alterations in the expression of two histone acetyltransferases (HAT1 and KAT2) which suggest that epigenetic events might participate in the regulation of nutrient effect on milk fat synthesis (Li et al., 2013).

Daughters of cows fed a protein supplement during the last trimester of pregnancy conceived earlier in their first breeding season and had overall greater pregnancy rates, suggesting that changes in maternal nutrient status during late pregnancy influenced the reproductive performance of the daughters (Martin et al., 2007). In another study, Sullivan et al. (2009) observed that heifers born to dams fed a diet high in protein during the second trimester of pregnancy had a decreased number of antral follicles at 2 years of age. In pigs, the effect of dietary protein restriction and excess during pregnancy was shown to alter epigenetic marks and the expression of key metabolic genes in offspring (Altmann et al., 2012, 2013). In weaning pigs, Cong et al. (2012) showed that a maternal low-protein diet during gestation and lactation affected hepatic cholesterol metabolism by modifying the epigenetic regulation of 3-hydroxy-3-methylglutaryl coenzyme A reductase and cholesterol-7alpha-hydroxylase genes, which suggest possible long-term consequences in cholesterol homeostasis later in adult life.

Nutrients also have effects on miRNA expression in farm animals. A high/low fat diet altered the miRNA expression in subcutaneous and visceral adipose tissues of cattle (Romao et al., 2012). In fact, a higher number of miRNAs were detected in the animals receiving the high fat diet as compared to the low fat diet (Romao et al., 2012). High producing dairy cows fed diets rich in unsaturated fatty acids produced a differentially regulated profile of miRNAs as compared to the same cows on control diets (Li et al., 2014a,b). These lines of evidence indicate that epigenetic marks respond to nutritional cues and thus have the potential to alter phenotypic outcomes.

It is evident that the nutritional composition of diets, how and when animals are fed impact how epigenetic marks drive gene expression and resultant phenotypes. Nutritional effects could be short or long term and more information is needed to determine when and how it can be effectively utilized in animal improvement. Harnessing the nutritional influence on epigenetic modulation of gene expression may positively impact livestock productivity. However, limited current knowledge restricts applicability thus highlighting the need to actively generate knowledge toward exploitation of the effects of nutrition on epigenetic marks toward improved livestock productivity.

## Epigenetic Marks and Livestock Health

Diseases caused by diverse agents including bacteria, viruses, parasites, and fungi are a major threat to livestock productivity worldwide and a leading cause to production losses. Although much effort has been put into understanding the mechanisms of livestock disease pathogenesis and control, complete eradication, or treatment still present major challenges. Understanding the contribution of epigenetic marks to disease parthenogenesis may provide further avenues of control.

In contrast to numerous reports of the involvement of epigenetic marks in the etiology of human diseases (Kulis and Esteller, 2010; Portela and Esteller, 2010; Rakyan et al., 2011), limited information exist on the role of epigenetic perturbations in livestock diseases. Following the findings of early studies in the 1970s and 1980s that showed that digesting thymus of bovine origin with trypsin and chymotrypsin enzymes resulted in the modification of the chromatin state and also that histone modifications and DNA methylation correlated with different states of the chromatin (Chatterjee and Walker, 1973; Lewis and Chiu, 1980), only a few studies to date have examined the relationship between the epigenetic state of cells and organs and the development of livestock diseases (Vanselow et al., 2006; Wang et al., 2013b). Investigating the involvement of epigenetic factors in bovine mastitis, the most common and costliest disease of dairy cattle, Vanselow et al. (2006) observed that a hypomethylated region of the upper promoter region of alpha S1 casein gene becomes remethylated (accompanied by shutdown of alpha S1 casein synthesis) following experimental challenge of the mammary gland with pathogenic *Escherichia coli* bacteria. This indicate that infection-related remethylation of this region remodeled the chromatin and spatially restricted regulatory mechanisms that protected the promoter against high levels of circulating prolactin and thus serve as an acute regulatory significance of CpG methylation (Vanselow et al., 2006). In the peripheral blood cells of clinical mastitic Chinese Holstein dairy cows, aberrant promoter methylation of the cluster of differentiation 4 (CD4) gene has been demonstrated, suggesting that the presence of bacteria changed the DNA methylation status of CD4 promoter in clinical mastitic cows (Wang et al., 2013b). Recently, the contribution of DNA methylation and histone acetylation to the control of bovine innate immune gene expression in relation to response to lipopolysaccharide (LPS) was demonstrated (Doherty et al., 2013; Green and Kerr, 2014). Exposure of dermal fibroblasts from dairy heifers which had previously displayed a differential response to LPS, demethylating 5-aza-2′-deoxycytidine (AZA) and hyper-acetylating trichostatin A (TSA) agents resulted in a loss of variability between individuals’ response to LPS. Treatment with AZA-TSA lead to altered expression of genes, including interleukin (IL)-8, IL-6, tumor necrosis factor alpha (TNF) and serum amyloid A3 suggesting an epigenetic regulation of LPS-induced responses and constitutive cytokine gene expression (Green and Kerr, 2014). In another study, LPS stimulation of peripheral blood mononuclear cells from healthy calves resulted in differential expression of *HDAC6*, *HDAC7*, and *DNMT3A* genes while treatment with the histone deacetylase inhibitor, TSA, significantly inhibited the expression of three pro-inflammatory cytokines [*TNF*, *IL2*, and interferon gamma (IFN)] thus suggesting an important role for the measured epigenetic enzymes in the regulation of bovine innate immune gene expression (Doherty et al., 2013). To determine the epigenetic mechanisms by which DNA methylation affects the function of bovine adaptive immune system during the peripartum period, Paibomesai et al. (2013) stimulated CD4+ *T*-lymphocytes from 5 Holstein dairy cows before and after parturition with concanavalin A (ConA) and from 3 Holstein dairy cows in mid-lactation with ConA alone or ConA plus dexamethasone and demonstrated significant effects on the expression of two cytokines, IFN-γ, type 1 and IL-4, type 2, which were also consistent with DNA methylation profiles of the IFN-γ gene promoter region but not with IL-4 promoter region. Recently, He et al. (2012) used a genome-wide approach to determine histone H3K27me3 modifications on blood lymphocytes in lactating Holsteins and reported a blueprint of bovine K3K27me3 marks that mediate gene silencing as well as indications that H3K27me3 plays its repressed role mainly in the regulatory region of bovine lymphocytes.

It is becoming increasingly clear that miRNAs play roles in bovine infection and immunity. A number of studies have shown that miRNAs are expressed in a wide range of bovine tissues including immune-related tissues (Coutinho et al., 2007; Xu et al., 2009; Hata et al., 2010; Vegh et al., 2013). A differential expression of four immune related miRNAs, miR-125b, miR-155, miR-146a, and miR-223 upon stimulation of bovine monocytes with LPS or *Staphylococcus aureus enterotoxin* B was demonstrated (Dilda et al., 2012). Similarly, Naeem et al. (2012) demonstrated a differential regulation of four miRNAs (Bta-miR-181a, miR-16, miR-31, and miR223) in bovine mammary tissue infected with *Streptococcus uberis* as compared to healthy tissue while Hou et al. (2012) showed that bta-miR-296, miR-2430, and miR-671 were up-regulated and miR-2318 was down-regulated in mammary tissues of cows with mastitis. Using next generation deep sequencing technologies, a number of studies have shown involvement of miRNAs upon viral and bacterial infections in bovine (Glazov et al., 2009; Lawless et al., 2013; Jin et al., 2014a). Upon viral (Bovine herpesvirus 1) infection of a cell line derived from adult bovine kidney and deep sequencing, Glazov et al. (2009) identified 219 known bovine and 268 novel miRNAs, some of which may be involved in animal’s response to the presence of the virus. Lawless et al. (2013) showed that 21 miRNAs were differentially expressed upon *S. uberis* infection of bovine primary epithelial cells. Similarly, Jin et al. (2014a) demonstrated a differential expression of nine miRNAs (bta-miR-184, miR-24-3p, miR-148, miR-486, and let-7a-5p, miR-2339, miR-499, miR-23a, and miR-99b) upon challenge of MACT-cells (bovine mammary epithelia cell line) with heat inactivated *E. coli* and *S. aureus* bacteria. These studies revealed unique miRNA profiles in response to Gram-positive and negative bacteria (Lawless et al., 2013; Jin et al., 2014a).

In Pigs, Tao and Xu (2013) studied miRNA expression during weaning stress and showed involvement of differentially expressed miRNAs in small intestinal metabolism, stressful responses and immune functions. Ye et al. (2012) examined miRNA expression in the duodenum of *E. coli* F18-sensitive and -resistant weaned piglets and identified 12 candidate miRNA (ssc-miR-143, ssc-let-7f, ssc-miR-30e, ssc-miR-148a, ssc-miR-148b, ssc-miR-181a, ssc-miR-192, ssc-miR-27b, ssc-miR-15b, ssc-miR-21, ssc-miR-215, and ssc-miR-152) disease markers. From lung tissue of pigs infected with *Actinobacillus pleuropneumoniae*, Podolska et al. (2012) identified miR-664-5p, miR-451, and miR-15a as promising miRNA candidates involved in response to bacterial infection.

Detailed knowledge on how different pathogens of importance in livestock production direct epigenetic modifications and effects on the expression of disease phenotypes may guide informed decisions on the development of strategies to effectively manage livestock diseases. This calls for coordinated efforts to make available the epigenome maps of different immune cell types in livestock species for exploitation for improved animal health.

## Epigenetic Marks and Regulation of Lipid Synthesis and Milk Production

The adipose tissues and mammary glands are the main organs that produce fat, a major form of energy storage. In addition, mammary glands of ruminant animals produce milk which contributes enormously to the nutrition of humans. Milk and fat are important economic traits in livestock productivity and the proper functioning of the mammary glands and adipose tissues is vital for desired productivity. A role for epigenetic marks in the growth and differentiation of these organs including lipid metabolism and adipogenesis has been demonstrated (Devinoy and Rijnkels, 2010; Fernández-Hernando et al., 2011; Rijnkels et al., 2013). Furthermore, there is growing evidence that epigenetic factors regulate milk production in dairy cows (Singh et al., 2010, 2012). As compared with a wealth of data on humans and mice, epigenetic regulatory roles in lipid synthesis in livestock (cattle, goat, sheep, and pig) mammary glands and adipose tissues as well as milk production are still scarce.

Pioneering work on the characterization of miRNAs in bovine adipose tissues and mammary glands led to the identification of 59 distinct miRNAs and initial clues of the involvement of these molecules in mammary gland functions (Zhiliang et al., 2007). Recently, the miRNA expression profile in bovine adipose tissues was characterized and about 20% were identified as being correlated with back fat thickness (Jin et al., 2010). In another study, high expression of two out of 15 specific miRNAs detected in fetal and adult back fat in cattle suggested roles in the development and maintenance of bovine subcutaneous fat tissue (Sun et al., 2014). Furthermore, functional analysis revealed that fat enriched miRNAs targeted genes with modulatory functions in lipid and fatty acid metabolism while muscle enriched miRNAs targeted cysteine and glycine-rich protein 3, a gene with function in skeletal and muscular system development (Sun et al., 2014). Comparing miRNA expression of muscle and adipose tissues, Li et al. (2012c) reported a great diversity of miRNA composition and expression levels between the two tissues and suggested a complex regulatory network may underlie subcutaneous fat development in pigs. A number of fat-deposition-related miRNAs were identified in castrated pigs suggesting important roles in fat deposition after castration (Bai et al., 2014; Cai et al., 2014). Examination of DNA methylation in adipose and muscle tissues of pigs showed that differentially methylated regions in gene promoters were highly associated with the development of obesity through repression of both known obesity-related genes and novel genes (Li et al., 2012d). Recently, Baik et al. (2014) observed that DNA methylation status regulated tissue-specific expression of adipogenic and lipogenic genes in the intramuscular fat and *longissimus dorsi* muscle tissue in Korean cattle.

At the level of the mammary gland, differences in types and expression levels of miRNAs have been reported between lactating and non-lactating bovine mammary glands (Li et al., 2012a). miRNA expression is affected by stage of lactation and also associated with genes across diverse biological pathways in bovine mammary glands (Wang et al., 2012). Additionally, a number of miRNAs including miR-148a, miR-26a, miR-21-5p, miR-27b, miR-143, bta-miR-30a-5p, let-7a-5p, let-7f, miR-10b, and miR-99a-5p are highly expressed in bovine mammary gland/mammary epithelial cells (Li et al., 2012a, 2014a; Jin et al., 2014a; Le Guillou et al., 2014) suggesting roles in the lactation process and mammary gland functions. In goat mammary glands, differential miRNA expression was detected between peak lactation and dry period, and between early and late lactation (Ji et al., 2012; Li et al., 2012b). Comparative analysis of the miRNA repertoire in lactating and non-lactating bovine and mouse mammary glands observed that 6 (miR-126-5p, miR-16-5p, miR-141-3p, miR-200a-3p, miR-200b-3p, miR-200c-3p) out of 24 miRNAs common to both species were highly expressed in lactating than non-lactating mammary glands (Le Guillou et al., 2014). In addition to detecting miRNA expression in the mammary gland, functional studies have directly linked several miRNAs with mammary gland physiology. Through target prediction analysis, growth hormone receptor (GHR) was determined to be targeted by miR-15a and functional analyses with a mammary epithelial cell line confirmed that miR-15a inhibited the expression of caseins, epithelial cell number as well as the expression of GHR mRNA and protein (Li et al., 2012e). Another miRNA, miR-103, was shown to control milk fat accummulation in goat mammary gland during lactation (Lin et al., 2013). A role for endogenous miRNA-143 in the differentiation of bovine intramuscular fat was demonstrated whereby transfection of fibroblast-like preadipocytes with miRNA-143 antisense inhibitor suppressed differentiation followed by decreased storage of lipid droplets and expression of key adipocytes regulatory genes such as CCAAT/enhancer binding protein-a and fatty acid binding protein-4 while miRNA-143 inhibitor transfection increased cell proliferation (Li et al., 2011a).

Based on these indications, it is obvious that epigenetic marks regulate lipid synthesis and milk production. It now remains to be determined how epigenetic factors can be managed to improve milk/meat quality like increasing the concentrations of desired fatty acids (e.g., conjugated linoleic acid) in milk and muscle tissues. This will be facilitated by a cataloging of the effects of epigenetic marks on these traits under specific conditions.

## Epigenetic Marks and Animal Reproduction

Pressure for improving economically important livestock traits has increased tremendously and this increase has been associated with declining fertility (Garnsworthy et al., 2008). As a result, assisted reproductive technologies including *in vitro* embryo production and somatic cell nuclear transfer/cloning are widely applied to enhance reproductive efficiency in farm animals (Pontes et al., 2010; Urrego et al., 2014). However, the developmental competence of embryos produced by these technologies differs greatly from their *in vivo* produced counterparts. Accumulated lines of evidence indicate that assisted reproductive technologies possibly interfere with imprint establishment/maintenance during gamete or pre-implantation embryo manipulation as seen by numerous reports of epigenetics implication in nuclear reprogramming deficiencies in cloned embryos (Bourc’his et al., 2001; Dean et al., 2001; Urrego et al., 2014). It was reported that majority of embryos derived through nuclear transfer die during post implantation development (Cibelli et al., 2002). These prompted investigations into methylation reprogramming in cloned embryos with the observations that demethylation and *de novo* methylation process are not properly accomplished in cloned embryos as compared to an active paternal demethylation of the genome shortly after fertilization followed by passive demethylation of the maternal genome during normal embryonic development (Dean et al., 2001; Yang et al., 2007). In the same light, a failure of histone modification reprogramming has been demonstrated in cloned bovine embryos (Santos et al., 2003) as well as differential histone 4 (H4) acetylation in the blastomeres of cloned bovine (Maalouf et al., 2008). Compared with normal fetuses, Couldrey and Lee (2010) observed subtle DNA methylation abnormalities in cloned fetuses in mid-gestation. Cloned animals are therefore plagued with such problems as respiratory complications, hepatic complications, large offspring syndrome, and placental dysfunctions (Meirelles et al., 2009). Consequently, assisted reproductive technologies are known to be responsible for a portion of the epigenetic disturbance during development (Meirelles et al., 2014; Urrego et al., 2014).

miRNAs also play important regulatory roles in livestock reproductive processes including ovarian function, follicular development, estrous cycle, fetal development, embryonic development, and spermatogenesis (Hossain et al., 2009; Su et al., 2010; Tripurani et al., 2010; Lian et al., 2012; Salilew-Wondim et al., 2014). Observations regarding miRNA regulation of the mammalian female reproductive system have been summarized by Carletti and Christenson (2009). miRNA expression in the bovine and pig ovary has been characterized (Hossain et al., 2009; Tripurani et al., 2010; Li et al., 2011b) and recently, Salilew-Wondim et al. (2014) highlighted the miRNA expression patterns of granulosa cells in subordinate and dominant follicles and their likely involvement with follicular recruitment, selection, and dominance during the early luteal phase of the bovine estrous cycle. Further roles for miRNAs in reproduction include prevention of early granulosa cell differentiation (Lei et al., 2010), mediation of granulosa cell responses to transforming growth factor b1 in pre-antral follicles and oestradiol production (Yao et al., 2010; Xu et al., 2011), support of granulosa cell survival during ovulation (Carletti et al., 2010), inhibition of anti-angiogenic factor expression during luteogenesis (Otsuka et al., 2008) and regulators of the follicular–luteal transition (McBride et al., 2012).

Elucidation of the role of epigenetic marks on observed effects following application of assisted reproductive technologies show that these technologies perturb normal developmental processes of the offspring. Such information is crucial as it will determine the conditions under which these technologies should or should not be used.

## Epigenetic Regulation of Growth and Development

Epigenetic modifications in mammals have essential and important roles in genome reprogramming and in the expression of genes that control growth and development. The phenomenon of gene imprinting, a process regulated by epigenetic mechanisms has been shown to regulate a wide range of biological processes including fetal growth and development, metabolism, and behavior (Jiang et al., 2007; Bartolomei, 2009; Lambertini et al., 2012). It is well appreciated that genomic imprinting plays physiological roles in metabolism and body composition throughout life and as such contributes to the typical variation and architecture of complex traits (Smith et al., 2006; Casellas et al., 2009; Hager et al., 2009). How imprinted genes influence livestock phenotypes has been a subject of active research in the past decade and recently, Imumorin et al. (2012) and Magee et al. (2014) summarized the effects of epigenetic marks on imprinted gene control of livestock growth and development, and productivity. Another recent review expatiated specifically on the epigenetic consequences of artificial reproductive technologies to the bovine imprinted genes, small nuclear ribonucleoprotein, H19-imprinted maternally expressed transcript/insulin-like growth factor 2 (IGF2), and insulin-like growth factor 2 receptor (IGF2R) (Smith et al., 2015). Furthermore, recent research continues to provide evidence of imprinted gene control of growth and development of livestock species (Couldrey et al., 2014; Huang et al., 2014a,b; Jin et al., 2014b). Examination of genome wide DNA methylation status of fetal and adult *longissimus dorsi* muscles of Chinese Qinchuan cattle revealed a negative correlation between methylation and expression patterns of high-read genes from nine different tissues at multiple developmental stages (Huang et al., 2014a). In different development stages in cattle, intragenic DNA methylation status was shown to down regulate the expression of IGF2 gene (Huang et al., 2014b). Furthermore, genome-wide DNA methylation changes have been reported in sheep muscle (Couldrey et al., 2014) and in skeletal muscle between young and middle-age pigs (Jin et al., 2014b). In another study, examination of the effect of maternal diets, consisted of either low-starch (haylage) or high-starch (corn silage) during gestation showed differential expression of three imprinted (H19, maternally expressed 8, IGF2R) and DNMT3a genes in *longissimus dorsi* muscle in calves between the diet groups (Wang et al., 2015). These findings indicate that epigenetic factors play critical roles in the expression of imprinted genes, cellular processes and the development of muscle tissue in livestock species.

## Transgenerational Epigenetic Inheritance in Livestock Species

Transgenerational epigenetic inheritance cannot be deliberated without first elaborating on germline reprogramming. Reprogramming is required to remove epigenetic signatures acquired during development or imposed by the environment so that subsequent elaboration of the body plan in the embryo properly reflects the genetic blueprint characteristic of each species (Heard and Martienssen, 2014). Transgenerational epigenetic inheritance occurs when reprogramming fails or is bypassed enabling the stable transmission of epigenetic marks (e.g., DNA methylation, histone modification, etc.) acquired in one generation to the next (Heard and Martienssen, 2014). Given the fact that epimutations can be transmitted from generation to generation, there is interest to determine how alterations of the epigenotype might underwrite the development of desired phenotypes in future generations. The best known example of mammalian transgenerational epigenetic inheritance occurs at the agouti viable yellow gene in the mouse. The DNA methylation state or epialleles of intracisternal A particle (IAP) retrotransposons inserted upstream of the agouti gene controls the expression of this gene (Daxinger and Whitelaw, 2010). IAP belongs to a small group of long terminal repeat retrotransposons that appear to resist germ line reprogramming in the gametes and early embryos of the agouti mouse leading to a range of coat colors from yellow, yellow and brown patches to brown according to degree of DNA methylation (Rakyan et al., 2003; Popp et al., 2010). In addition, several reports indicate that environmental influences such as exposure to chemicals, nutrition and maternal behavior cause modifications in gene expression that persist throughout life and may be transmitted to the next generation (Cooney et al., 2002; Weaver et al., 2004; Anway et al., 2005; Kaminen-Ahola et al., 2010).

Despite reports in previous sections that show effects of nutrients, disease pathogens and other factors on epigenetic marks in farm animals, only one study has examined transgenerational epigenetic response in a farm animal species (Braunschweig et al., 2012). The effects of dietary methylating micronutrients on gene expression and DNA methylation in a three generation Large White pig indicated significant differences in gene expression between groups and in DNA methylation at the promoter of the iodotyrosine deiodinase gene in F2 generation (Braunschweig et al., 2012). There are no further reports of epigenetic transgenerational effects in other domestic animals to date. The occurrence of transgenerational epigenetic inheritance is important for animal breeding purposes (Feeney et al., 2014) and more research is warranted to established transgenerational epigenetic effects and application in livestock production.

## Potential Application of Epigenetics Information in Livestock Production

### Rationale

As outlined in sections above, epigenetic marks induced by a wide variety of factors such as nutrition, maternal care, disease conditions, and stress exert enormous influence on the genome through effects on gene expression and phenotypic outcome under different conditions. Present day gains in livestock traits are the result of interaction between improved management practices (including how animals are fed, bred, reared, and managed), the genome and the environment that ultimately work in concert to determine resultant phenotypes. Furthermore, use of genomic information in selection is finding wide application in livestock improvement schemes (Wiggans et al., 2011; Bouquet and Juga, 2013) and obtaining genotype data has become a highly prioritized research area. The accuracy of genomic selection also known as genomic estimated breeding values (GEBV) is based on the association between genotypes, phenotypes, and pedigree information (Meuwissen et al., 2001; Dekkers et al., 2010). Already, application of GEBV has caused changes in most dairy breeding schemes with potentials for pig breeding including selection of animals at a young age and shortening of generation intervals (Hayes et al., 2009; Dekkers et al., 2010; Wiggans et al., 2011). Fewer young bulls are subjected to progeny testing than before due to the accuracy of pre-selecting on the bases of GEBV than on the basis of parent average breeding values and sires selected at an early age with the use of GEBV realized the full potential of genomic selection (Buch et al., 2012a,b). Thus, the use of genomic information in selection schemes has led to enhanced productivity and may also lead to more sustainable and profitable breeding schemes. It is, however, also well accepted that genomic information alone does not account for all of the heritable variation in livestock traits. Already, it has been shown that stable epigenetic markers can be used as prognostic tools for certain phenotypic traits in humans (Flintoft, 2010) and health intervention strategies based on epigenetic markers are already being attempted in humans (Nebbioso et al., 2012; Boese et al., 2013). The action of epigenetic marks coupled with their intricate relationships is transforming our understanding of gene regulation and effects on livestock traits. There is therefore no doubt that epigenetic regulation could have profound implications for the development of livestock traits of interest such as health, reproduction, development, behavior, nutrition, and milk production. Epigenetic mechanisms could be the missing yet uncovered players in the expression of complex animal production traits and disease etiology and are likely responsible for a portion of the missing variation in production traits and possibly for a portion of the heritability that is not accounted for in existing genetic assessment schemes. In discussing how epigenetic control the development and expression of quantitative traits, Jammes et al. (2010) opined that the consideration of epigenetic regulation in genetic evaluations could impact in two ways: (1) remove epigenetic bias in predicting an animal’s true genetic transmitting ability and (2) more accurately account for an important source of phenotypic ‘epigenetic’ variation that would reduce the number of progeny (for sires) and the number of dam and daughter records required for reliable estimates of an animal’s true genetics.

To account for epigenetic contribution to an individual’s true breeding value, consideration should be given to redefine the information used in calculating GEBV. Thus it is expected that, the accuracy of an individual’s GEBV would increase if estimated associations would include both genomic and epigenomic information. However, such epigenetic markers need to be stable or show evidence of being transgenerationally inherited. Alternatively, understanding how certain triggers, such as diets or management strategies modulate epigenetic marks could lead to improved management practices for increased productivity.

### Challenges

With advances in DNA sequencing technologies, the genomic portion of the variation in livestock traits is being assessed at an increasing rate in recent years. The epigenomic portion is not being given the attention that it clearly deserves. Epigenomic mutations, as is the case with DNA mutations, can have enhancing, deleterious or neutral attributes and have the potential to adapt and respond to environmental cues with great impact on heredity and breeding. Effects of epigenetic regulatory mechanisms must, however, be accurately analyzed to determine applicability. Unfortunately, epigenetic research activities on farm animal species are currently limited as compared to extensive work in humans and model organisms. This is partly due to insufficient recognition, limited tools, shortage of funding and lack of a global network of researchers.

A major limitation to livestock epigenomics research is insufficient recognition of the importance of epigenomic contributions to the emergence of livestock phenotypes of economic importance and disease traits. In time past, there was uneasiness regarding the value and usefulness of mapping the epigenomes of different cell types in humans (Madhani et al., 2008). Then, epigenomic variations like histone modifications were simply seen as a mirror of the activities of transcription factors, so assaying these modifications in different cell types would not offer useful/applicable information. However, concerted efforts from several research groups (Jones and Martienssen, 2005; Feinberg, 2007) led to the production of the epigenome maps of different cell types in humans (Rivera and Ren, 2013). The use of these maps has furthered our understanding of the contribution of the epigenome to different biological processes. The importance of producing the epigenome maps of the different cell types, organs and tissues of livestock species is no longer contestable.

In humans, tools that facilitate epigenetic research have been developed enabling the elucidation of epigenetic contributions to the development of disease traits. Supported by significant funding and involvement of a large global network of research teams, human epigenome maps (DNA methylation, chromatin modification state, and chromatin structures) have been generated (Rivera and Ren, 2013) enabling the development of assays that support both small scale and genome wide epigenetic studies. These maps have enabled investigations to provide further insights into how diverse factors alter epigenetic states in different organs and tissue types leading to the appearance of assorted phenotypes. Thus, more understanding of the mechanisms of human development including phenotypic variations among human populations, etiology of diseases, and effect of environmental insults have been gained through profiling of the epigenome. It is now known that even though the genome of the over 200 cell types in the human body are the same, it is the epigenome that serves to instruct unique gene expression patterns among the different cell types in response to different cues and at different stages of development. Therefore, recognition of the contribution of the epigenotype to phenotypic outcomes in livestock species and improved funding may attract people with specialized skills to animal epigenetic research and thus lead to discovery and application of epigenetic marks in animal production.

Recently, Couldrey and Cave (2014) reviewed the tools that could be used to assess DNA methylation levels in livestock species and concluded that a great deal of work is required before present technologies can find wide applications in animals. Due to the limitation of tools to support small scale studies, only a few genes at a time are considered in livestock epigenomics (DNA methylation, chromatin modification) studies while only limited studies have assayed specific cell types or muscles at a genome wide scale (He et al., 2012; Couldrey et al., 2014; Huang et al., 2014a). Also, limited technical knowhow in informatics management of large scale data generated by new sequencing technologies and platforms may slowdown epigenomics discovery in livestock species. Therefore, tools for livestock epigenetics research are needed to drive discovery and application.

### Opportunities for Progress

The application of epigenomic information in livestock breeding will depend on the availability of the genome sequence of the species in question. Sequencing of the bovine, pig, sheep, and goat genomes were completed recently (Elsik et al., 2009; Groenen et al., 2012; Dong et al., 2013; Jiang et al., 2014) and availability of several cow (Bovine UMD3.1 and BosTau1-8), pig (susScr2 and susScr3), sheep (oviAri 1 and oviAri 3) and goat (CHIR V1.0 and CHIR V1.1) genome builds are aiding genomic research and will also greatly facilitate epigenomic research in these species.

Advances in next generation sequencing technologies and falling cost of sequencing have enabled advances in genomic research and in epigenomic research in humans and mice. It is expected that with the right funding, these tools could be used to generate epigenomic information in livestock species and thus identify epigenetic markers that can be included in livestock improvement programs. Epigenome wide approaches have enabled mapping of epigenomics components in many cell types with millions of putative regulatory elements identified (Zentner and Henikoff, 2015). Advances in technology are making possible the functional characterization of these components. Recently, an epigenome editing technology based on CRISPR-Cas9 (clustered, regularly interspaced, short palindromic repeat–CRISPR-associated protein) was used to functionally direct effectors to specific well characterized histone modifying domains with the result that modulation of histone modifications leads to robust and specific transcriptional outcomes (Hilton et al., 2015; Kearns et al., 2015). The ability to target specific epigenomics regulatory elements may provide powerful tools for manipulating gene regulation. Epigenome-wide association studies (EWAS) from mouse and human studies have been performed on complex diseases (Rakyan et al., 2011). EWAS can complement large-scale genome-wide association studies (GWAS) which are on-going for livestock animals. GWAS identify single-nucleotide polymorphisms or other genetic variants that are associated with particular phenotypic traits. The variants identified by GWAS usually account for only a fraction of the total trait heritability. The ‘missing heritability’ can be explained in part by the epigenetic effects on gene regulation through EWAS studies (Doherty et al., 2014). Furthermore, advances in RNA sequencing technologies are enabling miRNA discovery in livestock species and the role of miRNA regulation of livestock phenotypes is beginning to emerge.

**FIGURE 2:**
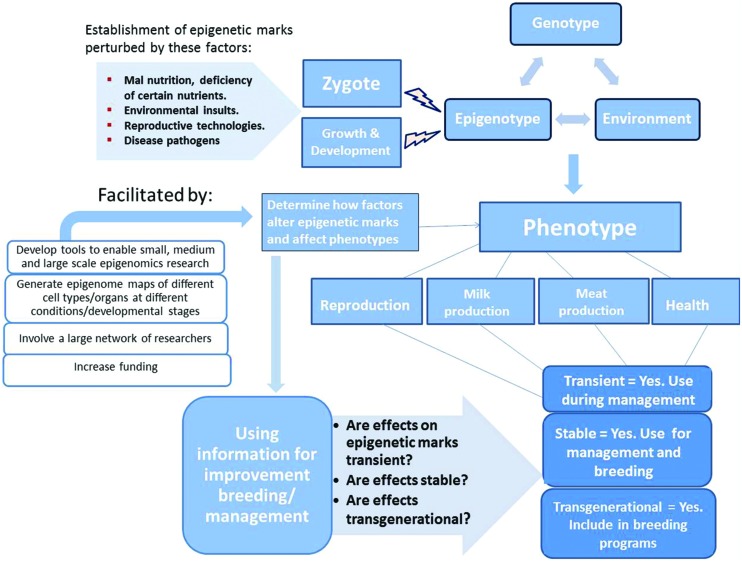
**Increasing evidence shows that the phenotype results from interaction of the genotype, epigenotype and environmental forces**. Establishment of the epigenotype can be perturbed during the zygotic stage (maternal environment) and during growth and development by several forces. The effect of such forces on epigenetic marks and influence on the phenotype needs to be recognized and determined before application in improvement breeding/management.

Breed improvement progress achieved through traditional animal breeding methodologies over the years has relied on selection on the basis of the phenotype. The phenotype as it is now known results from interaction of the genotype, epigenotype, and environmental/other factors (**Figure 2**). Therefore, both genomic and epigenomic information might have been unintentionally applied in animal breed improvement all along. Given overwhelming evidence that epigenetic marks contribute to the appearance of different phenotypes in livestock species, it is probable that the primary goal over the next decade will be to accelerate epigenetic research to enable the understanding of how epigenetic marks influence livestock phenotypes under different conditions. It is only then that epigenomic information can complement genomic information and provide a better understanding of the forces that shape livestock phenotypes and directional application in breed improvement and management practices (**Figure 2**).

## Conclusion

It is evident that epigenetic marks including DNA methylation, histone modifications, and non-coding RNAs contribute to regulation of biological processes in livestock with direct and indirect effects on reproduction, growth, health, and traits of economic importance. Appreciably, different genetic and epigenetic variants interact with environmental factors (e.g., nutrients, pathogens, etc.) to define individual variations and phenotypic outcomes. Advances in genomics technologies have made possible inclusion of genomic information in present day breeding programs. Genomic information alone does not account for all the phenotypic variations in livestock traits. Accumulating evidence continues to associate epigenetic marks with different phenotypic outcomes in livestock species pointing to the notion that unexplained phenotypic variation in livestock traits could be due to epigenetic factors. It is expedient to accelerate research on how epigenetic marks influence livestock complex disease and production traits under different conditions and include this information to increase animal productivity and sustainability.

## Conflict of Interest Statement

The authors declare that the research was conducted in the absence of any commercial or financial relationships that could be construed as a potential conflict of interest.
